# One novel *HSD17B4* mutation in association with D-bifunctional protein deficiency: a case report and literature review

**DOI:** 10.3389/fped.2025.1689571

**Published:** 2026-01-06

**Authors:** Lu Xiong, Shiqing Wang, Hui Sun, Tao Zhong, Li Li, Haijiang Zeng, Yubo Huang

**Affiliations:** 1 Department of Neonatology, Ganzhou People's Hospital (Ganzhou Hospital of Nanfang Hospital, Southern Medical University), Ganzhou, China; 2 Department of Neonatology, Southern Medical University Nanfang Hospital, Guangzhou, China

**Keywords:** D-bifunctional protein deficiency, HSD17B4, hypotonia, neonate, seizures

## Abstract

**Background:**

D-Bifunctional protein, also called D-peroxisomal bifunctional enzyme which is encoded by *HSD17B4* gene located in chromosome 5q21, catalyzes the second and third steps of preoxisomal β-oxidation of fatty acids and fatty acid derivatives. When HSD17B4 gene mutations cause varying degrees of decline in DBP function, it can lead to D-Bifunctional protein deficiency(D-BPD) which is a rare autosomal recessive discord. The typical symptoms include hypotonia and seizures.

**Case presentation:**

A 4-day-old female infant was admitted due to recurrent seizures for 3 days. Main clinical manifestations included facial dysmorphism, poor responsiveness, hypotonia, feeding difficulties, refractory seizures, bilateral hearing impairment, and an electroencephalogram (EEG) showing focal sharp waves generalizing to widespread discharges. Whole-exome sequencing revealed a homozygous mutation in the *HSD17B4*gene originated from her parents: Exon6: c.344A>T (p.Asp115Val), a variant not previously reported. During her hospitalization, she received respiratory support, nasogastric feeding and antiepileptic treatment. One month after discharge, telephone follow-up revealed frequent recurrent seizures, the parents of the patient refused further treatment due to poor prognosis and financial constraints.

**Conclusions:**

This article presents a case of a newborn who presented with hypotonia, feeding difficulties and refractory epilepsy shortly after birth, and was eventually diagnosed with D-bifunctional protein deficiency through whole-exome sequencing. The prognosis of this disease is poor, and symptomatic and supportive treatment is the main approach. Therefore, whole-exome sequencing is particularly important for definitive diagnosis when neonates present with generalized hypotonia, feeding difficulties and refractory epilepsy. In addition, a missense mutation [c.344A>T (p.Asp115Val)] is a newly discovered variant that deserve further study.

## 1 Introduction

Peroxisomal diseases comprise a heterogeneous spectrum of rare, inherited metabolic disorders, with an estimated prevalence of 1 in 25,000 to 50,000 live births (1–4). Peroxisomal diseases are categorized into two groups: peroxisome biogenesis disorders and single peroxisomal enzyme deficiencies. In patients with single enzyme deficiencies, peroxisome structure remains normal; the metabolic disorder(s) result exclusively from a functional impairment of a single enzyme protein within the peroxisome. D-Bifunctional protein deficiency (D-BPD),which belongs to single peroxisomal enzyme deficiencies, is a rare autosomal recessive disorder first reported in 1989 (5), with an estimated incidence of 1/100,000. D-bifunctional protein (DBP), which participates in the β-oxidation of very-long-chain fatty acids (6), is a peroxisomal enzyme that consists of three catalytic domains: an N-terminal 2-enoyl-CoA hydratase, a central 3-hydroxyacyl-CoA dehydrogenase, and a C-terminal steroid carrier protein 2-like domain (SCP-2L). Pathogenic mutations in the *HSD17B4* gene located at 5q21 are responsible for D-BPD. Based on the specific deficient enzymatic activity, D-BPD is categorized into four types. Type I is characterized by a combined deficiency of both dehydrogenase and hydratase activities. Type II presents with an isolated hydratase deficiency, whereas Type III features an isolated dehydrogenase deficiency. Type IV, which presents a less severe phenotype compared to types I–III, shares similarities with Perrault syndrome (PRLTS). Patients with D-BPD manifest a severe clinical course, marked by neonatal hypotonia, recurrent epileptic seizures, and a constellation of dysmorphic facial features. These features commonly include macrocephaly, a prominent forehead, hypertelorism, a flat nasal bridge, and low-set ears. The deficiency of one or both(hydratase or dehydrogenase) enzyme activity results in the impaired catabolism of very long-chain fatty acids (VLCFAs), dihydroxycholestanoic acid (DHCA), trihydroxycholestanoic acid (THCA), and pristanic acid. Consequently, the accumulation of VLCFAs, DHCA, and THCA serves as a hallmark biochemical manifestation of D-BPD. This can be diagnostically confirmed through functional assays and mutational analysis of the relevant enzymes in patient-derived cells, typically cultured skin fibroblasts (7). However, the definitive diagnosis of D-BPD is ultimately confirmed by genetic sequencing. D-BPD follows a rapidly progressive trajectory, with mortality typically occurring within the first two years of life. To date, only five cases have been reported in China (2, 8, 9), indicating limited diagnostic experience. This article presents a neonate with D-BPD caused by an *HSD17B4*gene mutation and reviews relevant literature to enhance pediatricians' understanding of the disease and guide early diagnosis.

## 2 Clinical data

### 2.1 Medical history

The patient was a four-day-old female infant admitted for recurrent seizures over three days. She was the second child of a non-consanguineous couple, delivered via cesarean section at 39 weeks and 1 day of gestation with a birth weight of 2.55 kg (<10th percentile). The mother had regular prenatal checkups without abnormalities. The parents and their healthy 12-year-old daughter were healthy. The family history was unremarkable for neurological or inherited metabolic diseases. Apgar scores were 9 at 1 min and 10 at both 5 and 10 min. Shortly after birth, she exhibited poor responsiveness, generalized hypotonia, and decreased oxygen saturation, prompting transfer to the neonatal intensive care unit (NICU) at 10 h of life for low-flow oxygen and antibiotic therapy. At 13 h of life, she developed seizures characterized by unilateral limb clenching and jerking, lasting 10–20 s and resolving spontaneously. Phenobarbital (25 mg IV) and midazolam (1 μg/kg/min IV infusion) were administered without achieving seizure control. Frequent apnea episodes necessitated transfer to our hospital. Upon admission, seizures recurred more than 10 times daily, manifesting as facial flushing, unilateral limb clenching, jerking, and facial muscle twitching, alternating between sides (predominantly right), lasting seconds to minutes. Respiratory distress with oxygen desaturation occurred independently of seizures.

### 2.2 Physical examination

**Figure 1:**
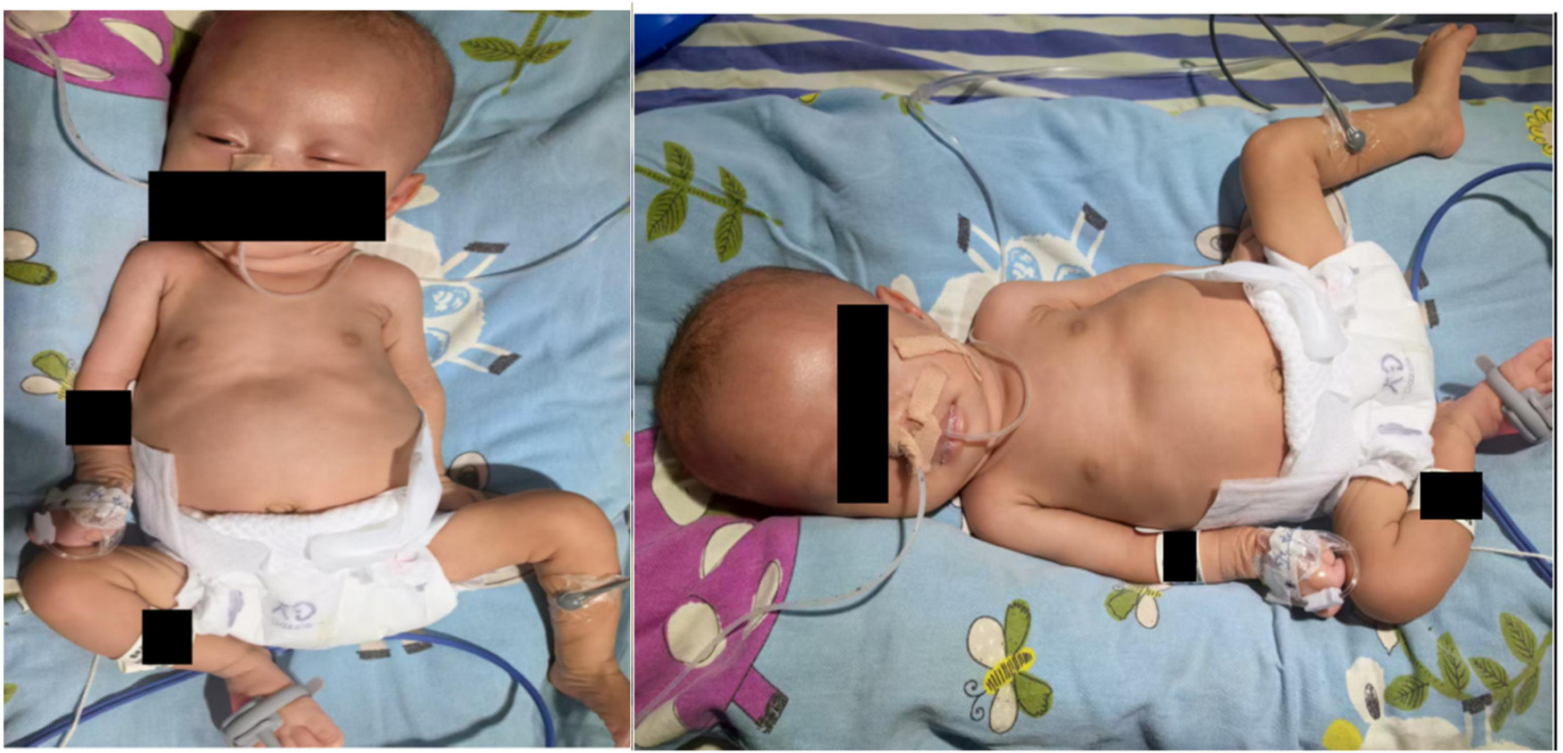
High forehead, wide anterior fontanelle, indifferent gaze, generalized hypotonia.

Vital signs: temperature 36.8 °C, pulse 142 bpm, respiratory rate 44/min, oxygen saturation 94% on non-invasive ventilation (FiO2 30%). Head circumference: 32.5 cm, length: 50 cm. Notable findings included a high forehead, wide anterior fontanelle, indifferent gaze, normal pupillary reflexes, weak cry, shallow and irregular respirations, coarse breath sounds, normal heart sounds, soft abdomen, generalized hypotonia, positive scarf sign, and absent primitive reflexes (Figure 1).

### 2.3 Laboratory and imaging findings

**Figure 2:**
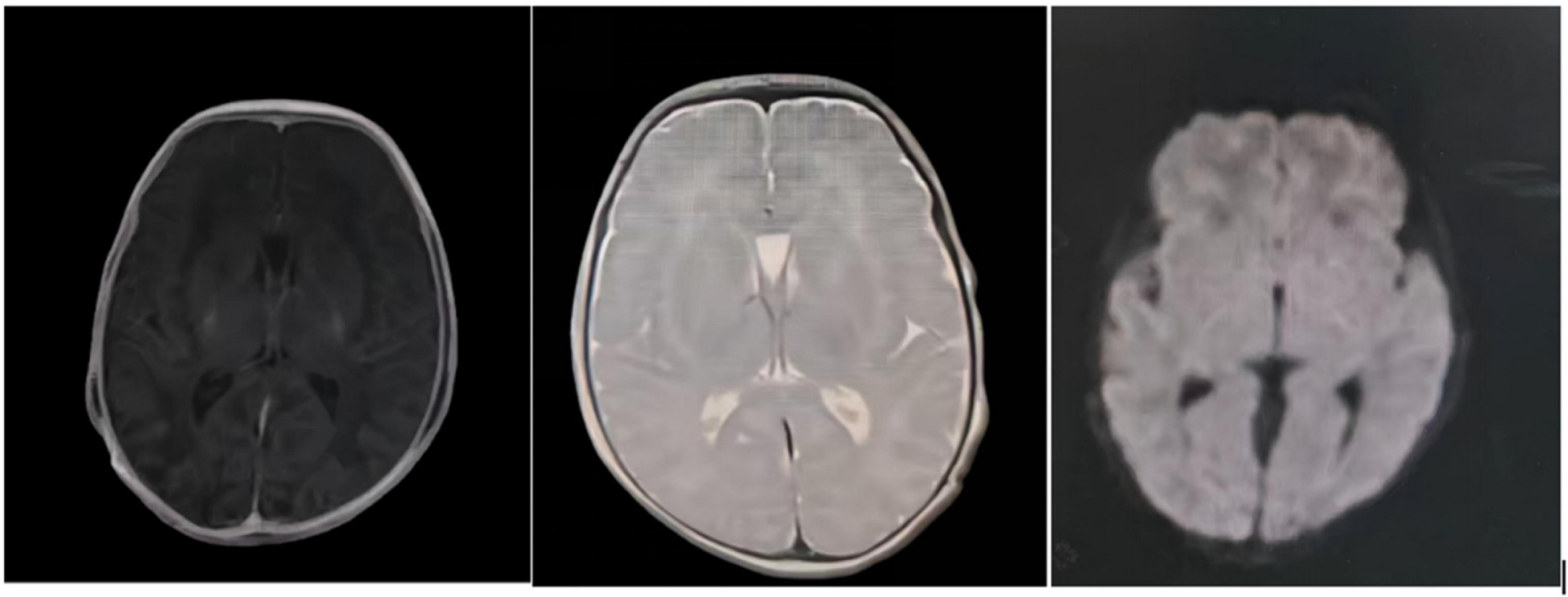
Magnetic resonance image at day 2 revealed normal.

Initial tests (day 1 to 2)at the local hospital (blood gas, complete blood count (CBC), liver/kidney function, electrolytes, C-reactive protein (CRP), procalcitonin (PCT), interleukin-6 (IL-6), infectious serology, thyroid function, cultures, and tandem mass spectrometry were unremarkable except for mildly low 25-hydroxyvitamin D (19.39 ng/mL). Chest x-ray suggested neonatal pneumonia (Based on the clinical presentation suggestive of a viral infection, antimicrobial therapy was withheld. Subsequent observation indicated that this initial diagnosis was not the cause of the patient's desaturation). Echocardiography revealed a patent foramen ovale (1.6 mm), patent ductus arteriosus (2.9 mm), and mild tricuspid regurgitation. Cranial MRI (day 2) was normal (Figure 2). Cerebrospinal fluid analysis (day 4) at our hospital was normal. Initial EEG (day 5) was normal for age, but subsequent EEG showed sharp waves in bilateral frontal-central-midtemporal regions, non-rhythmically distributed, without consistent clinical correlation.

### 2.4 Genetic testing

**Figure 3:**
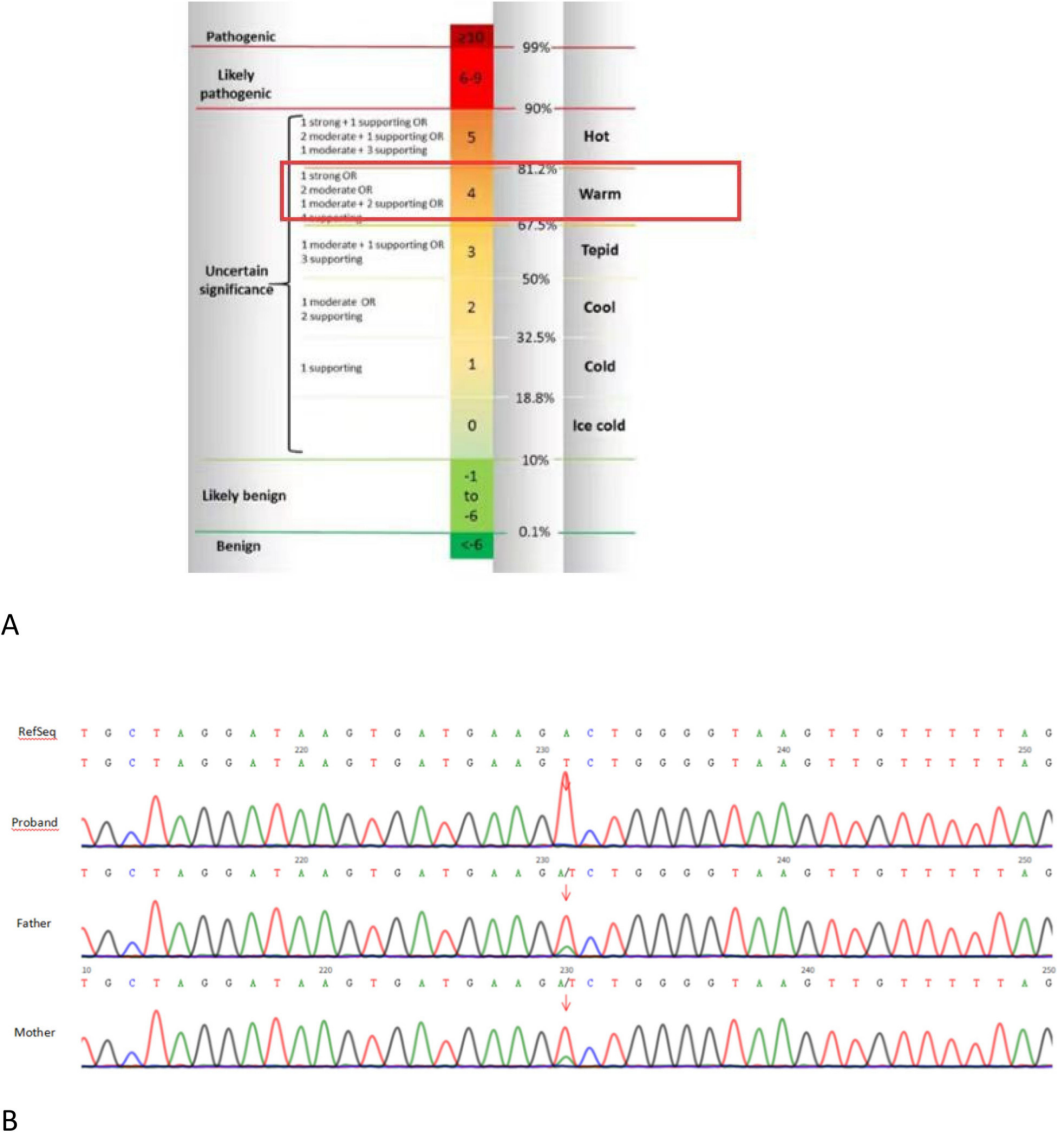
**(A)** The variant c.344A>T detected in the gene indicates “Variants of Uncertain Significance (VUS)”; **(B)** validation and source anaysis by whole-exome sequencing suggested that the homozygous pathogenic variant of the proband originated from the parents.

Whole-exome sequencing (performed by Nanchang ADICON Clinical Laboratory) identified a homozygous missense mutation in HSD17B4 (NM_000414.4: Exon6: c.344A>T, p.Asp115Val). The missense mutation c.344A>T is located in Exon 6 and may lead to impaired dehydrogenase function. This mutation is absent in HGMD (http://www.hgmd.cf.ac.uk) and ClinVar (http://www.ncbi.nlm.nih.gov/clinvar) databases. Evidence for Pathogenicity Classification: PM2_Supporting: The variant was absent from both the gnomAD population database and our local cohort. PP3_Moderate: Computational evidence from the REVEL algorithm supported a deleterious effect, with a predictive score of 0.919. PM3_Supporting & PP4: The proband was found to be homozygous for the c.344A>T variant and presented with a clinical phenotype consistent with D-BPD. According to the 2015 American college of Medical Genetics and Genomics(ACMG) guideline (10), the variant c.344A>T detected in the gene indicates “Variants of Uncertain Significance (VUS)” (Figure 3A). Peripheral venous blood was collected from the proband's parents for validation and source anaysis by whole-exome sequencing. The results suggested that the homozygous pathogenic variant of the proband originated from the parents (Figure 3B). Both parents of the proband were identified as heterozygous carriers of this missense variant and were asymptomatic. Parental testing confirmed the variant is consistent with an autosomal recessive inheritance pattern. Pedigree analysis could not be performed, as consent for further genetic testing of additional family members was not obtained. Consequently, we were unable to further corroborate the pathogenic status of this variant through familial studies.

### 2.5 Treatment course

**Figure 4:**
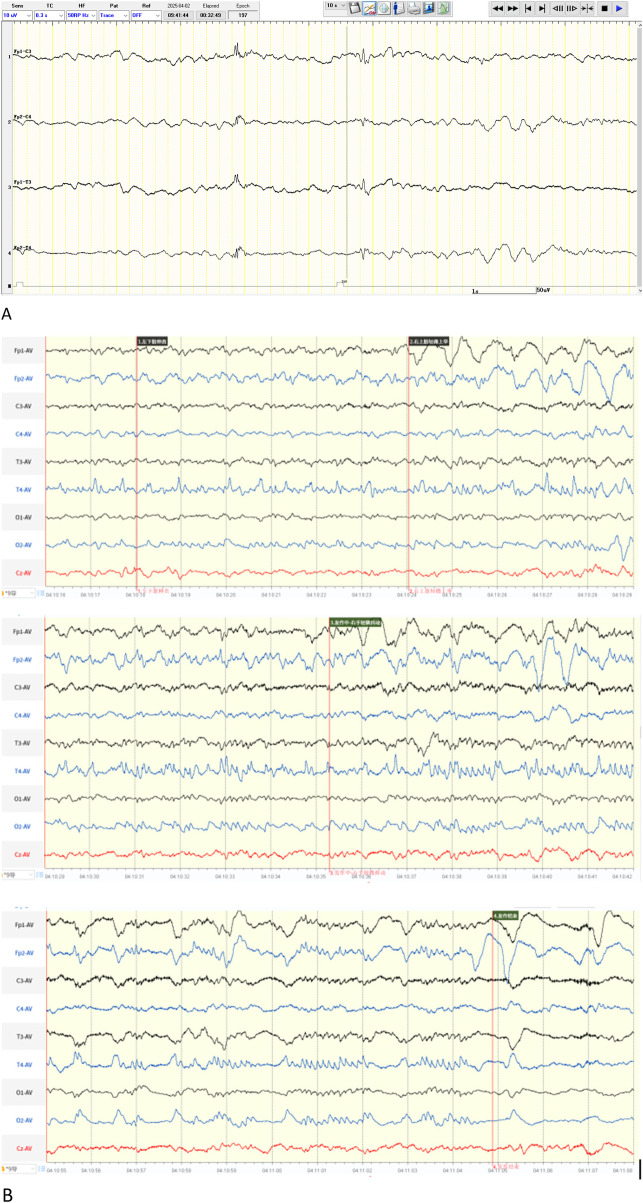
**(A)** aEEG at day 5 show sporadic sharp waves or continuous non-rhythmic short bursts in bilateral frontal, central, and midtemporal regions. **(B)** Repeat EEG(day 25) showed a background characterized mainly by diffuse slow-wave activity. During the interictal period, irregular sharp waves and sharp-slow waves were observed in bilateral temporal regions. Ictally, focal sharp waves originating in the central or temporal regions gradually generalized and progressed to widespread discharges.

The patient developed seizures 13 h after birth. After intravenous phenobarbital at the local hospital failed to control seizures, midazolam was administered via continuous IV infusion at 1 μg/kg/min. Her condition worsened with frequent apnea in addition to persistent seizures, prompting transfer to our department. Upon admission, we observed that seizures and apnea occurred at different times. Considering midazolam's potential respiratory depressant effect, it was discontinued. aEEG showed (Figure 4A) sporadic sharp waves or continuous non-rhythmic short bursts in bilateral frontal, central, and midtemporal regions, suggestive of ongoing seizures. Phenobarbital was administered intravenously with a loading dose of 20 mg/kg, followed by maintenance doses of 5 mg/kg every 12 h to sustain therapeutic levels. Repeat EEG showed no abnormal discharges. The phenobarbital blood concentration was 27.3 μg/mL, prompting a switch to oral administration (5 mg/kg q12h). Unfortunately, at 25 days of age, the patient experienced recurrent tonic-clonic seizures, accompanied by nystagmus, lip cyanosis, and transcutaneous oxygen saturation dropping to approximately 20%. Seizure duration exceeded previous episodes, lasting about 1–2 min, with a frequency of approximately 20–30 per day. Repeat EEG (Figure 4B) showed a background characterized mainly by diffuse slow-wave activity. During the interictal period, irregular sharp waves and sharp-slow waves were observed in bilateral temporal regions. Ictally, focal sharp waves originating in the central or temporal regions gradually generalized and progressed to widespread discharges. Intravenous phenobarbital (20 mg/kg) was administered twice, followed by IV maintenance (5 mg/kg q12h). This reduced seizure frequency to about ten times per day, without significant change in seizure type or duration. Subsequently, levetiracetam (7.5 mg/kg q12h PO) was added. Seizures improved compared to before: the nature remained unchanged, but duration significantly shortened and occurred only after external stimulation. At 31 days of age, whole-exome sequencing results reported a novel homozygous HSD17B4mutation [c.344A>T (p.Asp115Val)], suggestive of D-BPD. Further diagnostic tests (serum very-long-chain fatty acids, fibroblast biopsy) were declined by parents due to poor prognosis and financial constraints. One month after discharge, telephone follow-up revealed frequent recurrent seizures. A clinic visit was recommended but declined by the parents, resulting in loss to follow-up.

## 3 Discussion

D-Bifunctional protein (DBP) is a peroxisomal enzyme comprising three domains: 2-enoyl-CoA hydratase, 3-hydroxyacyl-CoA dehydrogenase, and a C-terminal steroid carrier protein 2-like domian(SCP-2L). It participates in the second hydration reaction and the third dehydrogenation reaction in the β-oxidation of very-long-chain fatty acids, branched-chain fatty acids, bile acid intermediates, and di/trihydroxycholestanoic acids (11). D-Bifunctional protein deficiency (D-BPD), caused by mutations in the HSD17B4gene, results in varying degrees of decline in DBP expression and activity. HSD17B4maps to 5q21, with 24 exons encoding distinct enzyme domains: the 3-hydroxyacyl-CoA dehydrogenase domain (exons 1–12), the central 2-enoyl-CoA hydratase domain (exons 12–21), and the C-terminal sterol carrier protein 2-like domain (SCP-2L) (exons 21–24) (1, 12, 13). D-BPD is classified into: Type I: Combined hydratase and dehydrogenase deficiency; Type II: Isolated hydratase deficiency; Type III: Isolated dehydrogenase deficiency (14). Recently, a milder Type IV with Perrault syndrome-like features has been proposed (4). Base on previous studise, in fibroblast samples from nearly all type I-deficient patients, DBP was undetectable by immunoblotting with an antibody against human DBP. Among type II-deficient patients, the 45 kDa hydratase unit was found to be absent in 90% of cases. The full-length protein and the 35 kDa dehydrogenase unit were detectable in 68% of this patient group. In type III deficiency, the 35 kDa unit was retained in 59% of patients, and the full-length protein together with the hydratase unit were identified in 96% of cases (7). This finding directly accounts for the most rapid disease progression and the shortest mean survival time(6.9months) observed in patients with type I D-BPD. However, a novel subtype (type IV) of D-BPD was proposed by McMillan et al. This category is defined by mutations that lead to functional deficiencies in both the dehydrogenase and hydratase domains. This contrasts with type I, as the enzymatic activities in type IV are not abolished but are present at markedly reduced, detectable levels. These findings collectively indicate that the *in vivo* function of DBP is more complex than previously appreciated, and warrants further investigation. A limitation of this is that no enzyme activity testing was performed in this case. However, based on the genetic result locating the mutation within exon 6, Type III deficiency is considered most likely.

To date (April 2023, http://www.hgmd.cf.ac.uk), 107 *HSD17B4*mutations have been reported, including 69 missense/nonsense, 3 splicing, 11 small deletions, 2 small insertions, 20 gross deletions, 1 gross insertion/duplication, and 1 complex rearrangement. The missense mutation identified in this patient is a novel mutation in *HSD17B4* [c.344A>T (p.Asp115Val)]. Ferdinandusse et al. (7) reported that the main clinical manifestations of neonates with D-BPD include craniofacial dysmorphism, hypotonia within the first month, and refractory epilepsy. The clinical manifestations of the patient reported here conform to these characteristics. Refractory epilepsy is a hallmark of neonatal-onset D-BPD. Seizures typically manifest as tonic-clonic or focal motor seizures involving unilateral limbs; facial twitching is less common. EEG often shows altered background activity and multifocal paroxysmal discharges. Most patients require high-dose polytherapy (≥3 antiepileptic drugs) for seizure control (9, 13, 15–17). The seizure characteristics and EEG findings in this patient were consistent with previous reports. However, during hospitalization, conventional doses of phenobarbital combined with levetiracetam achieved partial seizure control. This may be related to the relatively milder phenotype associated with Type III D-BPD. Furthermore, the patient was lost to follow-up shortly after disease onset, preventing assessment of long-term progression. Notably, this patient exhibited low 25-hydroxyvitamin D levels shortly after birth. Related reports are scarce. Werner et al. (14) reported a case of rickets in a child with D-BPD during follow-up. At 6 months, despite adequate vitamin D intake, the child presented with multiple fractures and significantly deficient serum 25-hydroxyvitamin D, attributed to bile acid synthesis defects impairing fat-soluble vitamin absorption. The early deficiency observed in our patient suggests a risk for later vitamin D deficiency rickets. To date, a total of five pediatric cases of this disease have been documented in China (2, 8, 9). All affected individuals presented with hypotonia and convulsive seizures during the neonatal period. Among these, two sibling cases exhibited neonatal convulsions; however, the seizures were not refractory and thus did not prompt clinicians to pursue whole exome sequencing for definitive diagnosis. Notably, the mutation profiles in these two cases are indicative of type I D-BPD. In contrast to previous reports associating this subtype with mortality before two years of age, both children survived into childhood. The underlying reasons for this discrepancy warrant further in-depth investigation.

It is noteworthy that there is accumulating evidence reporting patients with D-BPD who present with mild symptoms and a slow disease progression. Consequently, these individuals often remain undiagnosed until early juvenile or even adulthood when whole exome sequencing is ultimately performed. This article presents a review and comparison of previously published case reports, focusing on their clinical manifestations, laboratory results, and subtypes based on the time of diagnosis (Table 1). Reviewing the literature and Table 1, we found that the vast majority of neonates presenting in the neonatal period experience seizures and hypotonia, characterized by convulsions, limb rigidity, and possible loss of consciousness. Concurrently, some patients exhibit dysmorphic features (15–21), prompting clinicians to consider genetic disorders and pursue early genetic testing for diagnosis. In contrast, patients diagnosed during childhood or adulthood typically present with developmental delay and hearing impairment as initial symptoms, with a slowly progressive course. Early detection is often challenging, leading to longer diagnostic delays. Some of these patients may retain normal cognition and intelligence. Therefore, clinicians should remain vigilant for D-BPD in patients presenting with relevant clinical features. Whole-exome sequencing should be performed promptly for confirmation, enabling early intervention to improve quality of life.

**Table 1:** Comparison of clinical features, laboratory findings, and genetics in DBPD patients based on date of diagnosis (summarized from literature review).

Date of diagnosis	Adult (12, 22)	Juvenile (4, 23, 24)	Neonatal (14, 25, 26)	Present case
Clinical features				
Motor delay	Partially&initial	Partially&initial	+	+
Speech delay	Partially&initial	Partially&initial	/	/
Seizures	−	Partially	+	+
Hypotonia	−	+	+	+
Feeding difficulty	−	Partially	+	+
Hearing impairment	+	+	+	+
Intellectual disability	Partially normal	Partially normal	/	/
Ataxia	+	+	/	/
Dyscoordination	NR	+	/	/
Imaging				
Brain MRI(common image changes)	Cerebellar atrophy	Cerebellar atrophy	Corpus callosum hypoplasia/White matter lesions	Normal
Plasma metabolites				
Phytanic acid	Normal	Normal/↑	Normal/↑↑	NR
C26:00	Normal	Normal	↑↑	NR
C26/C22 ratio	Normal/↑	Normal	↑↑	NR
C24/C22 ratio	Normal/↑	Normal	↑↑	NR
Presumed DBPD type	II/III	II/III/IV	I/II/III	III

**Partially&initial**: Some patients have symptoms and these are the initial symptoms; *Partially:* Some patients have symptoms; **Partially normal**: A subset of patients had normal findings; +, exhibit the associated clinical features; −, not exhibit the associated clinical features; NR, not reported; /, not applicable; ↑, slightly elevated; ↑↑, significantly increased.

## 4 Conclusion

This report describes a novel *HSD17B4*missense mutation [c.344A>T (p.Asp115Val)] in a D-BPD patient, expanding the genetic spectrum. Whole-exome sequencing plays a crucial role in diagnosis. Prompt diagnosis is crucial for enabling genetic counseling and prenatal diagnosis, essential for reducing the incidence of birth defects. Meanwhile, for patients with residual enzyme activity, active symptomatic treatment can improve quality of life.

## Data Availability

The original contributions presented in the study are included in the article. Further inquiries can be directed to the corresponding author/s.
